# Participation by national parliaments in the EU legislative process

**DOI:** 10.1007/s12027-021-00675-5

**Published:** 2021-08-03

**Authors:** Adam Cygan

**Affiliations:** Leicester Law School, University of Leicester, Leicester, United Kingdom

**Keywords:** National parliaments, Subsidiarity, Legislative process, Legitimacy

## Abstract

Europeanisation has marginalised national parliaments and their democratic practices leading to a ‘de-parliamentarisation’ within the EU. The Treaty of Lisbon included substantive provisions designed to improve participation by national parliaments in EU decision-making, the most significant of which is the allocation of subsidiarity monitoring. This was intended to address concerns that national parliaments are peripheral within the EU Polity, and that EU legislation lacks legitimacy amongst its citizens. Protocols 1 and 2 of the Treaty of Lisbon promote a horizontal political dialogue between national parliaments within subsidiarity monitoring, but, experience of the last ten year indicates that this has not improved legislative legitimacy, nor adequately addressed de-parliamentarisation. This article argues that, while the Treaty of Lisbon has enhanced the privileges of national parliaments, they have not been ‘re-centred’ as an influential collective bloc of actors within the EU’s institutional framework.

European integration or ‘Europeanisation’ utilises a multilevel governance framework which has created a shared policy and legislative agenda which Member States are bound to implement.^1^ Through successive Treaties, legislative competences have been progressively transferred to the Council and European Parliament at the expense of national institutions and actors.^2^ In particular, the absence of direct national parliamentary participation in decision-making, together with the limited and often ineffective domestic accountability of the executive in EU affairs that have been identified as the primary cause of the democratic deficit within the EU’s legislative process.^3^ This isolation of national parliaments within the integration process has been more commonly referred to as the ‘de-parliamentarisation’ of the EU^4^ and is why national parliaments have been referred to as ‘victims’ of EU integration.^5^

However, the marginalisation of national parliaments within the EU legislative process may be more accurately defined as ‘de-national parliamentarisation’. The Treaty of Lisbon acknowledged this criticism by including a revised Protocol No. 1 on National Parliaments which extended the rights of national parliaments to directly receive EU documents and Article 12 TEU, which provides that ‘national parliaments contribute *actively* to the good functioning of the Union’ (emphasis added). This ‘active contribution’, for example through participation in subsidiarity monitoring under Protocol No. 2 on the Application of the Principles of Subsidiarity and Proportionality, creates an expectation that, collectively, national parliaments may inject enhanced democratic legitimacy into EU legislation. However, Protocol No. 2 has, arguably, failed to fully deliver the improved oversight intended with the subsidiarity Early Warning Mechanism (EWM) yielding only three subsidiarity objections to date since 2009 – the so-called ‘yellow cards’. Yet, notwithstanding this, it would be inappropriate to only measure the success of the EWM by these results.

The ‘active contribution’ of national parliaments has also manifested itself in other ‘softer’ ways with national parliaments taking greater control of their political responsibilities. By changing their *modus operandi* for both policy and legislative scrutiny, partly in response to the opportunities created by subsidiarity monitoring, there has been improved coordination between national parliaments through sharing information through forums such as the Conference of European Affairs Committees of National Parliaments (COSAC).^6^ Equally important to the formal provisions in Protocols No. 1 and No. 2 has been the development of political dialogue between national parliaments and the Commission. Though not formally within the Treaty of Lisbon it may be said that Protocols 1 and 2 have provided the impetus and incentive for national parliaments to become more proactive and self-assured institutions within the EU policy and legislative processes.

Parliaments may therefore be said to have become more ‘Europeanised’ in their behaviour and as a result of the Treaty of Lisbon developments^7^ the enhanced recognition of national parliaments within the Treaty provides a concurrent opportunity for both subsidiarity monitoring and improved oversight of ministers to their parliaments.^8^ To that extent this article considers the procedural opportunities for national parliaments to participate in EU decision-making and whether these opportunities for engagement can be said to have addressed criticisms of de-parliamentarisation and enabled national parliaments to engage in more effective participation in EU decision-making.

## National parliaments and the Treaty of Lisbon

The willingness to enhance the role of national parliaments in the EU decision-making was driven by two distinct factors. One is related to democratic norms and the other to process. Firstly, the long-held concerns associated to the popular legitimacy and the democratic quality of the EU fed the view that the EU system of governance should be more visibly ‘parliamentarised’.^9^ Though the increased legislative role of the European Parliament, which had since the Treaty of Amsterdam become a co-legislator with the Council, supported an argument of improved parliamentarisation it soon became clear that the parliamentarisation at the EU level was not sufficient and should be expanded to embrace more formally the national parliamentary institutions.^10^

This view was based both on the democratic quality and proximity given to national parliaments and on their capacity to identify and promote national interests and features in a period where the EU level was suspected of neglecting them. It was the Laeken Declaration^11^ that first placed the issue of the increased participation by national parliaments firmly on the EU reform agenda and this included a statement of the possibility that national parliaments could realise a ‘preliminary checking of compliance with the principle of subsidiarity’. This political scrutiny of the application of the principle of subsidiarity by the EU Institutions was a role which the Working Group IV of Convention on the Future of Europe recognised to be one which national parliaments, with their established practices and procedures of EU scrutiny were well placed to fulfil.^12^

## The principle of subsidiarity in EU law

The inclusion of Protocol No. 2 in the Treaty of Lisbon which allocated the task of subsidiarity to national parliaments was arguably the single most important development for national parliaments since their contribution was first recognised by Declaration 13 of the Treaty of Maastricht. There are three reasons for this. Firstly, subsidiarity monitoring confirms the presumption that, within a multi-level governance framework, legislation is made at the appropriate level of governance. Secondly, in order to maximise the effect of Protocol No. 2, national parliaments are expected to engage in a horizontal political dialogue, principally through COSAC. By so doing they should, as far as possible, seek to arrive at a consensus concerning the compliance of a legislative proposal with subsidiarity.^13^ But, this task is not without difficulties because, *inter alia*, the Treaty of Lisbon does not provide a clear and unambiguous explanation of what constitutes subsidiarity. Finally, recognition of national parliaments as guardians of competence acknowledges the presence and importance of the Nation State within EU integration. Parliaments, above all other institutions, provide a tangible embodiment of the State, and it is they who have dutifully ratified each new Treaty that has enabled deeper integration and transferred competences to EU institutions.

The principle of subsidiarity is contained within Article 5 TEU. Under Article 5 (3) TEU, in policy areas which do not come within the scope of exclusive EU competence^14^ the Union shall act only if, and ‘insofar as the objectives of the proposed action cannot be achieved by the Member States …but can rather by reason of scale or effects of the proposed action be better achieved at the EU level’. This, *prima facie*, may suggest a presumption against EU action, but, since the Treaty of Maastricht, the precise meaning of subsidiarity has been the subject of much debate.^15^ The Court has favoured a restricted interpretation,^16^ shared by the Commission, and, in whose view, Article 5 TEU raises a presumption that EU legislation is required if it satisfies the dual requirements of necessity and effectiveness.^17^

By contrast Wyatt^18^ has criticised that this dual requirement of necessity and effectiveness as being expansive because it is difficult to rebut, especially where the Commission proposes harmonising legislation for the regulation of the Internal Market. Wyatt has proposed an alternative single test;^19^ namely ‘can the objectives of the proposed action *only* be achieved through [EU] wide action’? If the answer to this question is ‘no’ then action should be taken at the national level. On this interpretation the purpose of subsidiarity monitoring would be to distinguish between those measures which *will* produce an EU wide outcome, from those which *must*.^20^ This distinction is of significance for parliaments because the single requirement would focus them on the core task of Protocol No. 2, i.e. did the EU need to act to attain the objective? Moreover, this does not automatically require consideration of related questions of proportionality within Article 5 TEU. Wyatt’s analysis also exhibits greater consistency with the allocation of input and output legitimacy post Lisbon. Once national parliaments determine that the EU should not act, it is, in his view, unnecessary to consider the effectiveness of legislation. Moreover, even if parliaments conclude that EU action is appropriate, this should also imply that they consider that the measure is proportionate.^21^

### Subsidiarity before the Court of Justice of the European Union

In *ex parte British American Tobacco*^22^ the Court considers what constitutes necessity and effectiveness for the purposes of Article 5 TEU. Weatherill has described this judgment as a ‘drafting guide’ for the proper scope of legislative harmonisation and by implication for the proper application of the subsidiarity principle.^23^ The Court’s judgment in *ex parte British American Tobacco* seeks to define the outer limits of EU competence to regulate the Internal Market and the Court stated that the EU does not, by virtue of Article 5 (3) TEU and the principle of subsidiarity therein, enjoy an exclusive competence to regulate *all* economic activity in the Internal Market.^24^ As such EU competences are limited to the elimination of trade and other barriers and to prevent the distortion of competition within the Internal Market. However, as Wyatt has noted where the Commission proposes legislation for this purpose it is very difficult for national parliaments to refute the Commission’s motives.^25^

This approach towards the need for EU legislative action expressed by the Court in the *British American Tobacco* judgment has subsequently been confirmed by the Court in its *Vodafone* judgment.^26^ In this case the Court was asked, *inter alia*, to consider whether an EU Regulation^27^ which set standard roaming charges for mobile phone users across the EU infringed the principle of subsidiarity and proportionality. The hub of the issue was how it could be determined that the measure was necessary to achieve the efficient functioning of the Internal Market and that differential prices across the Member States were not just a question of disparities between national rules regulating mobile phone operators. Central to the argument for EU legislative action was the presence of a genuine cross border element which, without EU legislation, would mean distortions in trade and competition. The Court held that the scope of the legislation was proportionate to protect consumers from high roaming charges. On subsidiarity grounds the Court justified EU legislative action on the basis that there was a genuine Internal Market in roaming services which could only be regulated through harmonising legislation.^28^ In its Impact Assessment that accompanied the legislative proposal the Commission concluded that individual legislation by the Member States would inevitably be heterogeneous in both substance and form and reinforce the division of markets.^29^ In particular, the Impact Assessment recognised that EU legislation was required to regulate a market that consisted of two interrelated parts, that is a wholesale market between mobile phone providers and a direct consumer market. In both markets the Court concluded that there was present a significant cross-border activity^30^ and these were inter-dependent upon each other. This, the Court concluded, necessitated a single common approach and comprehensive EU legislation which would guarantee the smooth functioning of the Internal Market.

Both the *British American Tobacco* and *Vodafone* judgments illustrate that subsidiarity, as is common with all regulatory principles, must be subject to judicial review. Judgments such as *Vodafone* illustrate that in the context of the ordinary legislative procedure the principle of subsidiarity creates regulatory expectations amongst both Member States and non-State actors with regard to which level of governance will legislate. This decision ultimately impacts upon the participatory and procedural prerogatives of national parliaments. The judgment in *Vodafone* primarily confirms what has been accepted for many years; namely that a Commission justification within an Impact Assessment that harmonising legislation is required is, as Wyatt argued, very difficult to rebut at the conclusion of a legislative process through the use of *ex post* judicial adjudication. Recognition of economic imperatives, the principle of market access and the need for effective consumer protection within an Impact Assessment and the discretion that the Commission exercises undoubtedly influenced the Court in *Vodafone* and other cases when faced with adjudicating on the application of subsidiarity. This would appear to reinforce the view that the oversight of the subsidiarity principle is probably most beneficial at the pre-decision stage of the law-making process. It should take place through effective political scrutiny by national parliaments which, albeit to only a limited extent, may exert *some* direct influence over national ministers and the EU Institutions, and is arguably more effective than *ex post* litigation before the Court.

## Protocols 1 and 2 Treaty of Lisbon

The Treaty of Lisbon provided the first formal legal recognition of the democratic significance of national parliaments in Article 12 TEU. It mentions national parliaments on several occasions in relation not just to their information rights which enable them to engage in subsidiarity monitoring but also their participation in the procedures of revision of the Treaty, their control over the field of Freedom, Security and Justice and their possibility to cooperate with each other and with the European Parliament.^31^ But it is the Treaty developments in Protocol No. 1 and Protocol No. 2 which introduced new prerogative and procedures related to the early control of the principle of subsidiarity which were held up as being of most significance for national parliaments.

Firstly, the inclusion of Protocol No. 1 on National Parliaments in the European Union provides for an extended right to receive a variety of documents *directly* from the Commission, or other originating institution. In a scrutiny process where time is of the essence this was a noteworthy development from the limited arrangements under the Amsterdam Protocol on national parliaments. Protocol No. 1 requires the direct provision of draft legislative acts,^32^ the annual legislative programme, Council agendas and minutes^33^ as well as the annual report of the Court of Auditors.^34^ Of most significance is the commitment to transmit draft legislative acts *directly* to parliaments which is a prerequisite for any ensuing subsidiarity monitoring. But the improved provision of information is only part of the picture; the provision of information must also be *timely*. The protracted provision of information had consistently proved problematic for national parliaments,^35^ primarily because their internal scrutiny mechanisms and the ordinary legislative procedure operate independently. Thus, an important development in Protocol No.1 is that it extended this period to eight weeks from the previous six week provided for under the Amsterdam Protocol, with a further ten days elapsing between placing the proposal on the agenda and its adoption under the ordinary legislative procedure.

Secondly, it is the inclusion of formal procedural guarantees to engage in scrutiny which were arguably the biggest development in the Treaty of Lisbon. In particular, the inclusion of subsidiarity Early Warning Mechanism (EWM) enables each assembly within each national parliament,^36^ to assess whether a legislative proposal made by the Commission infringes upon the principle of subsidiarity. If one third of the assemblies conclude that action at the EU level is not legitimate—the so-called ‘yellow card’—then the Commission is required to provide further justification. Protocol 2 also includes what is known as the ‘the orange card’ procedure which applies only to proposals under the ordinary legislative procedure and necessitates a simple majority of votes cast. The Commission may amend, maintain or withdraw the proposal, but, if it chooses to maintain the proposal, it must provide reasons for doing so. In these circumstances the European Parliament and Council must, before the conclusion of the First Reading, benchmark the proposal against the Subsidiarity criteria, taking in to account the views of the parliaments and the Commission. If the Council acting by a majority of 55%, or the European Parliament by a majority of the votes cast find against the proposal, then it will fall.

The EWM within Protocol 2 has created a formal framework for national parliaments to engage in a direct dialogue with the EU institutions which enables them to express politically significant reasoned opinions on the application of the principle of subsidiarity within the context of a specific legislative proposal. Commenting on the original provisions within the Constitutional Treaty, the House of Lords EU Committee Report concluded that “the raising of a yellow card would have a significant effect on the EU Institutions … if national parliaments operate the mechanism *effectively* [emphasis added] it would be hard for the Commission and the Council to resist such sustained political pressure”.^37^ However, ‘effectively’ implies the presence of a substantial consensus between national parliaments on the question of the application of subsidiarity and the procedural requirements of Protocol 2 do not presume or guarantee this consensus. It is this limitation that has proved to be the most significant challenge to the operation of Protocol 2.

### The early warning mechanism in action

The special rules related to subsidiarity checks have undoubtedly proved the most significant development for national parliaments. According to Protocol No. 1 and Protocol No. 2, national parliaments have eight weeks to deliver a reasoned opinion if they consider that draft legislation does not comply with the principle of subsidiarity. Each national parliament possesses two votes. In bicameral parliamentary systems, each of the two chambers possesses one vote and each chamber is entitled to issue reasoned opinions independently.

Under Protocol 2, if at least one third of national parliaments^38^ share the opinion that the draft legislation does not comply with the subsidiarity principle, it must be reviewed, therefore resulting in a “yellow card”. The threshold falls to one quarter for a draft legislative proposal submitted on the basis of Article 76 TFEU.^39^ After the ‘yellow card’ review, the authoring institution—which in the majority of instances will be the Commission—may decide to maintain, amend or withdraw the legislation. Legally, the procedure does not thus impose a binding requirement on the Commission to withdraw the legislative proposal. But in the case where the proposal is maintained or even amended, the procedure requires the Commission to provide a robust justification of why there is no infringement of the subsidiarity principle and explain the value-added effects of the legislative proposal.

Under the ordinary legislative procedure, if a simple majority of national parliaments consider that the draft legislative proposal does not comply with the principle of subsidiarity, the draft must be re-examined by the Commission, therefore resulting in an “orange card”. After such a review the Commission may decide to maintain, amend or withdraw the proposal. If the Commission decides to keep the proposal it must justify its position. The European Parliament and Council must then consider, before concluding the first reading, whether the proposal is compatible with the principle of subsidiarity. If the Parliament by a simple majority of its Members or the Council by a majority of 55% of its members consider that the proposal does not comply with the principle of subsidiarity, it is dropped. Specifically, in a case where a majority of national legislatures express concern on compliance with subsidiarity and are not heard by the Commission, it is uncontroversial for the Council or the European Parliament to reject the draft proposal.

The threshold of one third of the parliamentary assemblies has been reached only on three occasions since its inception. In 2012, a yellow card was raised for the first time^40^ on a proposed regulation on the exercise of the right to take collective action, the so-called ‘Monti II’ proposal^41^ which was the Commission response to the judgments in *Viking* and *Laval*.^42^ The Commission denied any breach of the principle of subsidiarity but decided to withdraw the proposal which it did in September 2012. Indeed, as the text required a unanimous agreement within the Council, the yellow card was perhaps also interpreted by the Commission as a signal of the likely opposition from some Member States within the Council.^43^

In 2013, a second yellow card^44^ was triggered in relation to the Commission’s proposal for a regulation establishing the European Public Prosecutor’s Office.^45^ If the reasoned opinions are reviewed it becomes evident that the assemblies were motivated by a variety of views, perhaps indicating an absence of collective agreement. Some assemblies, for example, the UK, Dutch and Irish Parliaments were opposed to the proposal and took an orthodox interpretation of subsidiarity as their position. This was legislation that should only be introduced by Member States. Others such as Hungarian and Romanian Parliaments were broadly in favour of the Public Prosecutor but concerned by the control of the Commission over it—which means that the notion of subsidiarity was arguably more relevant in their case. Others, including the Austrian Parliament, also used subsidiarity as a pretext as the proposal was not ambitious enough in their view. Faced with this patchwork, the Commission decided to leave the proposal unchanged. Yet, the final approved version of the legislation was substantially revised which would suggest that the scrutiny by national parliaments sent an indication to the EU institutions which led to them accepting significant amendments during the subsequent legislative procedure.^46^

Thirdly, in May 2016, a total of 14 parliamentary chambers objected to the Commission’s proposal for a revision of the Posted Workers Directive.^47^ This proposal generated significant responses from the national parliaments, particularly in Central and Eastern countries, as indicated by the parliaments raising opinions.^48^ The parliaments from those countries were therefore voicing national concerns, which their governments had already raised concerning the perceived disproportionate impact that the proposed legislation would have on their citizens to provide cross-border services if left unamended.

### The limits of Protocol 2

The primary effect of the institutionalisation and democratisation of the EU is that this has led to a progressive transfer of competence to supranational institutions for policies that were previously within the exclusive sovereign jurisdiction of Member States. In many cases the Treaty in Article 4 TFEU provides that competence is shared between the EU and the Member States leading to inevitable tensions concerning the application of these competences as illustrated through judgments such as *Vodafone*. This explains, in large part, why subsidiarity monitoring under Protocol 2 has been largely ineffective and only led to three yellow cards.^49^ For example, in circumstances involving the regulation of the Internal Market when competence is shared and the principle of subsidiarity is relevant, the determinant of EU action at the EU or national level is whether EU legislation is necessary and that the desired result can be more efficiently achieved through a single legislative measure. When the Commission makes such judgements, which should be evidence decisions based on quantitative and qualitative factors,^50^ this is then benchmarked against the principle of subsidiarity in Article 5 TEU. However, though the political judgment of national parliaments may decide against the need for EU legislation the Court has consistently held that once it is considered necessary to adopt common rules to regulate the Internal Market then this can *only* be achieved through EU action.^51^ Thus it may be argued that the reason for only three yellow cards is not necessarily that EU law is subsidiarity compliant, but rather that national parliaments have concluded that there is only limited value in the operation of Protocol 2.

The presumption underlying the Court’s reasoning has been that the legislative measure will comply with the principle of subsidiarity and, from a political accountability perspective this would appear to imply that national parliaments have, by and large, accepted the efficiency argument. However, this presumption is not without difficulty and while it may be procedurally correct to conclude that the legislative measure conforms to the principle of subsidiarity this does not immediately confirm that the legislation can be considered as legitimate. For example, notwithstanding the EU Better Regulation Agenda and the systematic use of Impact Assessments, concerns have been expressed that the reasons given by the Commission for EU legislative action tend to lack sufficient quantitative and qualitative justifications.^52^

This raises questions of when the EU does legislate whether it does so excessively and whether the legislative content also conforms to the principle of proportionality under Article 5 TEU. It is the inability of national parliaments to sufficiently reach agreement under Protocol 2 TEU, as the three yellow cards illustrates, that leaves open the possibility of the evolution of a newly formulated democratic deficit rising at the national level as a result of national parliaments not being to apply the principle of subsidiarity with consistency and consensus.^53^ This may be an unintended consequence, but Protocol 2 requires constant and coherent horizontal interparliamentary cooperation in order for it to be effective. While this cooperation may have improved, the evidence of only three yellow cards in 10 years strongly suggests that optimism about the constitutional potential of Protocol 2 were misplaced.

The absence of a ‘red card’ is considered within some parliaments as a weakness of the Early Warning Mechanism.^54^ The ability of parliaments to apply a brake to the legislative process would suggest real influence.^55^ Working Group IV of the Convention of the Future of Europe rejected the idea of national parliaments either individually or collectively curbing the legislative process as being inconsistent with democratisation and institutional balance.^56^ But in 2015, the question of a ‘red card’ returned in the form of one of the UK demands as part of its pre-referendum ‘re-negotiation’ that would enable national parliaments to block draft EU legislation. The proposal that the President of the European Council offered in February 2016 sought to make the EWM more efficient: 55% of national parliaments would be able to challenge a draft legislative act; it would then be discussed in the Council of the EU which would discontinue the consideration of the draft legislative act in question unless the draft was amended to accommodate the subsidiarity concerns of national parliaments. In reality the UK position was seeking to apply the principles of the ‘orange card’ to all EU legislative proposal. However, though the renegotiation became redundant after the result of the UK Brexit referendum the question of whether there should be a ‘red card’ for national parliaments has not wholly disappeared from the political agenda. For example, a discussion of the merits of a ‘red card’ was mentioned in a background note for the COSAC plenary in November 2016 that had been prepared by the Slovak Presidency Parliament.^57^

## The growth of political dialogue

The provisions of Protocols 1 and 2 should therefore not be viewed as a panacea which have unilaterally collectivised or improve engagement with subsidiarity monitoring. But these protocols may be identified as provide the motivation for a process of enhanced political dialogue between the Commission and national parliaments. This very point was recognised by then Commission President José Manuel Barroso who, in 2006, launched the eponymous Barroso Initiative in the aftermath of the rejection of the Constitutional Treaty which halted the implementation of the subsidiarity monitoring arrangements. Since its inception, the process of political dialogue between national parliaments and the Commission, and commonly referred to as the ‘Barroso initiative’ has, with some significant success,^58^ addressed the broader information and engagement asymmetries which Jans and Piedrafita have identified as the primary causes of de-parliamentarisation.^59^ One key development under the Barroso initiative is that it included, for the first time, the transmission not only of all legislative proposals but also policy consultation papers *directly* to national parliaments. Though these were not legislative proposals and therefore outside the scope of the EWM, they did provide national parliaments with an opportunity to move ‘upstream’ and communicate their priorities on EU policy proposals directly to the Commission before legislation was published.

The Barroso Initiative is underpinned by two objectives which place the convergence of parliamentary participation in EU affairs at the centre. First, it includes a commitment to national parliaments to provide a wider selection of documents that goes beyond legislative proposals and which reinforces the argument of a reduced reliance on Protocol 2. Secondly, the Initiative encourages both a vertical and a horizontal political dialogue with the Commission as well as between national parliaments. Protocols 1 and 2 remain important, if for no other reason that they provide legally binding guarantees within the EWM. But parliaments have, in practice, benefited more directly from the Barroso Initiative which encourages them to position themselves within the EU polity through a vertical political dialogue with the Commission. Accordingly, national parliaments have reacted positively to the Initiative and generated over 4,000 written opinions under the political dialogue process which have been sent to the Commission since 2006.^60^

The Treaty of Lisbon provisions and Barroso Initiative have repositioned national parliaments and moved them from the margins of EU decision-making and situated them more visibly within the EU polity. Crucially, both developments recognise the need for improved accountability secured through both political dialogue and institutional participation and notwithstanding the limited success of the EWM, overall national parliaments are, today, more prominent and self-assured actors in EU affairs. But it would be incorrect to conclude that the criticism of de-parliamentarisation has been fully addressed and criticism that the EU continues to suffer from a democratic deficit have not abated either.

National parliaments have welcomed the Barroso initiative and used it extensively, notwithstanding that the written opinions sent to the Commission are not reasoned opinions for the purposes of the EWM and so are not formal subsidiarity assessments. However, in the 2019 Annual Report for Dialogue between the European Commission and National Parliaments the Commission noted that it had only received 159 written opinions from national parliaments in the previous year under the political dialogue process.^61^ This is significantly lower than in previous years (576 in 2017^62^ and 569 in 2018^63^) and even fewer than in the previous transition year, 2015 (350).^64^ It is the lowest number since the beginning of the political dialogue in 2007. But 2019 was also the first year, since the introduction of EWM, in which the Commission received no reasoned opinion from national parliaments. This was largely due to the sharp decrease in the Commission’s legislative activity in the transition year between two Commissions and so it may be expected that in both cases national parliamentary activity will increase in 2020/21, especially as EU policy making and legislative activity increases in response to the Covid-19 pandemic.

## Concluding remarks

The prerogatives under the Treaty of Lisbon in Protocols 1 and 2 have offered improved opportunities for national parliaments to participate in the EU legislative process. Subsidiarity monitoring remains an important task that has been allocated to national parliaments, but as the evidence illustrates, the impact of the EWM is limited with no prospect on the horizon that there will be any significant improvement of the EWM thresholds being reached. While the increased transmission of EU documents from the Commission to national parliaments may reduce the information asymmetry between national parliaments and their governments and improve the scrutiny process, national parliaments’ ability to play a more active role in EU decision-making remains restricted. This is in large part due to the lack of coherent horizontal coordination and consensus amongst national parliaments with respect to subsidiarity monitoring. Going forward, the increased use of political dialogue may assist national parliaments, but this interaction is outside the scope of Protocol 2 and the EWM and only allows for an exercise of what may termed as ‘soft power’. If national parliaments are to become more ‘centred’ and have greater influence over the EU legislative process this will most probably require further reform which grants them additional privileges but such developments are unlikely to occur.

## Footnotes

1 *Moravscik* [16] at 22.

2 *Kassim* [12] at 297.

3 *Kiiver* [13] at 41–42.

4 *O’Brennan and Raunio* [17].

5 *Maurer* [14].

6 The Conference of European Affairs Committees of national parliaments, founded in 1989 is known by the acronym COSAC from its French title (*Conférence des organes spécialisés dans les affaires communautaires*). COSAC meets biannually in the Member State holding the Presidency of the Council.

7 *Goetz and Meyer-Sahling* [7].

8 *Kiiver* [13] at 48–49.

9 *Menon and Weatherill* [15] at 116.

10 *Maurer* [14] and FCC *Lisbon Treaty judgment* of June 20 2009 *Bundesverfassungsgerich*t, BVerfG, 2 BvE 2/08.

11 Laeken Declaration on the Future of Europe, 15 December 2001, available at: www.europa.eu.int/futurum/documents/offtext/doc151201_en.htm (last accessed 3 April 2021).

12 See CONV 353/02 Final Report of Working Group IV on the Role of National Parliaments at paragraphs, 4-8.

13 *Cooper* [2] at 447.

14 As included in Article 3 TFEU.

15 *Cooper* [1]; *Weatherill* [18]; *Gonzalez* [9].

16 Case C-58/08 *Vodafone* ECLI:EU:C:2010:321.

17 See COM(2008) 586 Final The Report of the European Commission on Subsidiarity and Proportionality 2007 at p. 2.

18 *Wyatt* [19] at 5.

19 *Wyatt* [19] at p. 6. See also the written evidence of Professor Wyatt to the House of Lords Select Committee on the European Union, HL 101 (2004-5) *Strengthening national parliamentary scrutiny of the EU—The Constitution’s early warning mechanism* at 4-5.

20 HL 101 (2004-5) at p. 3.

21 Under the Protocol the Commission is required to provide, *inter alia*, a financial and regulatory impact assessment of the proposed legislation.

22 Case 142/84 British American Tobacco ECLI:EU:C:1987:490.

23 *Weatheril* [18] at 834.

24 *Weatheril* [18] at 836.

25 *Wyatt* [19] at 7-8.

26 See Case 58/08 *Vodafone* at paragraphs 72-73.

27 Regulation No. 717/2007/EC on Roaming on Public Mobile Networks in the Community and Amending Directive 2002/21/EC.

28 See Case 58/08 *Vodafone* at paragraph 74.

29 See Case 58/08 *Vodafone* at paragraphs 75-76.

30 See *Weatherill* [18] at 832-4; *Horsley* [10] at 12-14.

31 Article 9 of Protocol 1 requires the European Parliament and national parliaments to determine together ‘the organisation and promotion of regular inter-parliamentary cooperation within the Union.’ Under Article 12 (f) TEU national parliaments and the European Parliament participate in ‘inter parliamentary cooperation in accordance with Protocol 1’.

32 See Article 2 of Protocol 1. This includes draft legislation that has originated from a group of Member States under Article 329 TFEU and proposals for policy coordination under Article 5 TFEU.

33 Article 5 of Protocol 1.

34 Articles 1 and 2 Protocol 1.

35 See CONV 353/02 at para. 14.

36 *Cygan* [3] at 486-7.

37 In evidence to the House of Lords Select Committee on the European Union former UK judge to the Court of Justice of the European Union, Sir David Edward, stated that if national parliaments did not raise Subsidiarity objections at the yellow card stage then it may be harder for any party to run subsidiarity arguments later. See HL 62-1 *The Treaty of Lisbon: an Impact Assessment* at paragraph. 11.40.

38 Post Brexit this now requires 18 of the 54 votes.

39 This covers proposals submitted under judicial cooperation in criminal matters and police cooperation.

40 The reasoned opinions submitted to the Commission was as follows: Belgian House of Representatives (1), Danish Parliament (1), Finish Parliament (1), French Senate (1), Latvian Parliament (1), Luxembourg Chamber of Deputies (1), Maltese House of Representees (1), Polish Sejm (1), Portuguese Assembly (1), Swedish Parliament (1), Dutch House of Representatives (1), British House of Commons (1).

41 COM(2012) 130: Proposal for a Council Regulation on the exercise of the right to take collective action within the context of the freedom of establishment and the freedom to provide services.

42 Case C-438/05 *International Transport Workers’ Federation and Finnish Seamen’s Union v Viking Line ABP* *and OÜ Viking Line Eesti* EU:C:2007:772 and Case C-341/05 *Laval un Partneri Ltd v Svenska Byggnadsarbetareförbundet, Svenska Byggnadsarbetareförbundets avdelning 1, Byggettan and Svenska Elektrikerförbundet* EU:C:2007:809.

43 For a fuller analysis of the events surrounding the first yellow card see Ian Cooper ‘A yellow card for the striker: national parliaments and the defeat of EU legislation on the right to strike’ (2015) 22 Journal of European Public Policy 1406-1425.

44 The reasoned opinions submitted to the Commission was as follows: Polish Senate (1), Romanian Chamber of Deputies(1), Romanian Senate(1), Dutch Senate(1), Dutch House of Representatives(1), French Senate (1), Hungarian National Assembly (1), British House of Lords (1), Cyprus House of Representatives (1), British House of Commons (1), Irish House of Oireachtas (1), Swedish Parliament (1), Maltese House of Representatives(1), Slovenian National Assembly (1).

45 COM/2013/0534 final. Proposal for a Council Regulation on the establishment of the European Public Prosecutor’s Office.

46 *Fromage* [5].

47 *Fromage and Kreilinger* [6].

48 The reasoned opinions submitted to the Commission was as follows: Czech Chamber of Deputies (1), Czech Senate (1), Polish Sejm (1), Polish Senate (1), Seimas of the Republic of Lithuania (2), Estonian Parliament (2) and the National Council of the Slovak Republic (2) Danish Parliament (2), Croatian Parliament (2), Romanian Chamber of Deputies (1), Romanian Senate (1), Latvian Saeima (2) Bulgarian National Assembly (2), Hungarian National Assembly (2).

49 *Goldoni*, [8].

50 This is part of the Commission’s broader Better Regulation Agenda. See COM(2005) 97 final Communication from the Commission to the Council and European Parliament—Better Regulation for Growth and Jobs in the European Union.

51 See Case C-49/01 *R v Secretary of State for Health ex parte British American Tobacco and Imperial Tobacco* EU:C:2002:741 in particular paras 177 and 185.

52 See for example the Interinstitutional Agreement between the European Parliament, the Council of the European Union and the European Commission on Better Law-Making, [2016] OJ L 123, 12.5.2016, p. 1–14.

53 See further *Cygan* [4] Chaps. 5 and 6.

54 See HC 563 (Session 2007-8) at p. 27.

55 There is one exception available under the Treaty where the equivalent of a’ red card’ may be applied by a single national parliament. This concerns decisions taken under Article 81 (3) TFEU which determine those aspects of family law with cross-border implications which may be the subject of acts adopted under the ordinary legislative procedure. Any proposal under Article 81 (3) TFEU must be notified to parliaments and any *one* may block it by indicating their opposition within six months.

56 See CONV 353/02 at para. 27.

57 This issue may be revisited as part of the consultations taking place in the Conference on the Future of Europe in 2021 where one of the themes includes how to strengthen democratic processes governing the EU. See COM(2020) 27 final Communication from the Commission to the European Parliament and the Council Shaping the Conference on the Future Of Europe.

58 *Jančić* [10].

59 *Jans and Piedrafita* [11].

60 COM(2018) 491 final. Annual Report 2017 on Relations Between the European Commission and National Parliaments. Available at https://ec.europa.eu/info/sites/default/files/law/law-making_process/documents/annual-report-2017-relations-between-european-commission-national-parliaments_en.pdf (last accessed 19 April 2021).

61 See COM(2020) 272 final. Annual Report 2019 on the Application of the Principles of Subsidiarity and Proportionality and on Relations with National Parliaments https://ec.europa.eu/info/sites/default/files/com-2020-272-en.pdf (last accessed 19 April 2021).

62 See COM(2018) 491 final at 61 above.

63 COM(2020) 272 final Report from the Commission Annual Report 2019 on the Application of the Principles of Subsidiarity and Proportionality and on Relations With National Parliaments.

64 COM/2016/0471 final Report from the Commission Annual Report 2015 on Relations Between the European Commission and National Parliaments.
